# Biochar and Hyperthermophiles as Additives Accelerate the Removal of Antibiotic Resistance Genes and Mobile Genetic Elements during Composting

**DOI:** 10.3390/ma14185428

**Published:** 2021-09-19

**Authors:** Yanli Fu, Aisheng Zhang, Tengfei Guo, Ying Zhu, Yanqiu Shao

**Affiliations:** Advanced Materials Institute, Qilu University of Technology (Shandong Academy of Sciences), Jinan 250014, China; f18766162009@163.com (Y.F.); zhangaisheng1207@163.com (A.Z.); gtf17663718837@163.com (T.G.)

**Keywords:** biochar, hyperthermophile, antibiotic resistance genes, bacterial community, compost

## Abstract

Sewage treatment plants are known as repositories of antibiotic resistance genes (ARGs). Adding biochar and inoculating with exogenous microbial agents are common ways to improve the quality of compost. However, little is known about the effects of these exogenous additives on the fate of ARGs during composting and the related mechanisms. In this study, municipal sludge was taken as the research object to study the ARG-removal effects of four composting methods: ordinary compost (CT), compost with hyperthermophiles (HT), compost with hyperthermophiles and 2.0% biochar (HT2C) and compost with hyperthermophiles and 5.0% biochar (HT5C). Real-time quantitative PCR (qPCR) and 16S rRNA high-throughput sequencing were conducted to analyze the ARGs, MGEs and bacterial community. After composting, the abundance of ARGs in CT was reduced by 72.7%, while HT, HT2C and HT5C were reduced by 80.7%, 84.3% and 84.8%, respectively. Treatments with different proportions of biochar added (HT2C, HT5C) had no significant effect on the abundance of ARGs. Network analysis showed that Firmicutes and Nitrospirae were positively associated with most ARGs and may be potential hosts for them. In addition, redundancy analysis further showed that the class 1 integrase gene (*intI1*), pH and organic carbon had a greater effect on ARGs. Our findings suggested that the combination of hyperthermophiles and biochar during the composting process was an effective way to control ARGs and mobile genetic elements (MGEs), thus inhibiting the spread and diffusion of ARGs in the environment and improving the efficiency of treating human and animal diseases.

## 1. Introduction

In recent years, the emergence of antibiotic resistance genes (ARGs) has become an important global health problem [1]. The number of deaths from antibiotic-resistant infections is estimated at 700,000 per year worldwide [2,3]. ARGs are ubiquitous and can be detected in river water, farm soil, sludge, vegetables and even the air [4]. In contrast to traditional pollutants, ARGs can transfer through mobile genetic elements (MGEs), spread between different species, and are heritable [5]. These ARGs may contaminate human drinking water and food, posing a serious threat to human health through the food chain.

Wastewater treatment plants are known as repositories of ARGs [6]. Municipal sludge is the focus of research on ARGs, MGEs and antibiotic-resistant bacteria (ARB). Simultaneously, this sludge contains a large number of nutrients and trace elements that are needed by plants. After harmless treatment, it can be applied to soil to effectively improve the soil structure and act as soil fertilizer to promote better growth of plants [7]. Aerobic composting is an effective method for sludge treatment and resource recovery in which organic matter in sludge is quickly decomposed by microorganisms and transformed into humus under aerobic conditions; at the same time, the harmful substances are removed or stabilized [8]. A massive number of studies indicated that composting has an important effect on the content of ARGs and is an effective method for reducing the abundance of ARGs [9]. Composting can reduce antibiotic resistance by altering the microbial structure and reducing the microbial load [10,11]. Some researchers found that most of the sulfonamide-, tetracycline-, quinolone- and macrolide-resistance genes were reduced in the compost [12]. Some researchers found that most tetracycline resistance genes tended to decrease but the abundance of some remained unchanged, while the abundance of others increased significantly during the composting process [13]. Although conventional composting showed some effectiveness in eliminating antibiotic residues and ARGs, it is worth noting that the persistence of ARGs and bacterial communities during conventional composting remains a serious threat. Hence, improving composting technology to effectively control antibiotic resistance is crucial.

Previous studies showed that adding exogenous additives into compost can improve the removal of ARGs. Some researchers found that zeolite that was added during composting reduced the total abundance of ARGs in the compost products [14]. Some researchers showed that the abundance of ARGs and MGEs decreased in the high-temperature period after adding bamboo charcoal and bamboo vinegar in the composting process [15]. Biochar, which is a kind of high-carbon material that is formed by high-temperature pyrolysis of biomass materials, is widely utilized in composting. It was shown that the rich functional groups and higher pore structure of biochar can improve the removal rate of ARGs [16]. Additionally, biochar can also improve the activity of microorganisms and the humification process [17], which also significantly impact the abundance of ARGs [18]. At the same time, prior studies have confirmed that the addition of biochar to compost has a crucial role in controlling bacterial communities [19,20]. These studies all confirmed that the addition of biochar to the composting process has an important effect on the abundance of ARGs.

The temperature was also shown to play a crucial role in the removal of ARGs during composting. It was observed that an ultra-high temperature composting process can effectively remove ARGs and MGEs from municipal sludge, and its removal rates were increased by 89.0 and 49.0% compared with ordinary composting [21]. The addition of hyperthermophiles to the composting process can increase the temperature, effectively reducing the abundance and diversity of most ARGs hosts [22]. The rapid increase in compost temperature depended on the addition of hyperthermophiles rather than on external heat sources [23]. To date, there is no data on the control of ARGs using the combined action of hyperthermophiles and biochar in the sludge composting process. The above studies show that the addition of biochar or hyperthermophiles alone has a significant effect in reducing ARGs in municipal sludge, and we hypothesized that the combined effect of both is more pronounced.

This study added hyperthermophiles and biochar to the composting process to simulate different composting conditions and explored the mechanisms of the effects of hyperthermophiles and biochar on ARGs and MGEs. The three kinds of resistance genes (*tet* genes, *sul* genes and *erm* genes), 16S rRNA and *intI1* were quantified using real-time quantitative PCR (qPCR). The changes in the bacterial community were determined using the 16S rRNA high-throughput technique. The main objectives of this study were as follows: (1) to investigate the effects of adding hyperthermophiles and biochar on the abundance of ARGs and MGEs in compost; (2) to investigate the effects of adding hyperthermophiles and biochar on the dynamics of microbial communities; (3) to determine the correlation between ARGs and potential host bacteria; and (4) to investigate the relative contributions of physicochemical properties, MGEs and bacterial communities to the changes in ARGs in compost. The research results of this study can be used to explore a more effective method to remove ARGs in the process of composting and improve the quality of composting products.

## 2. Materials and Methods

### 2.1. Materials and Experiment Setup

Municipal sludge was used as the raw material for compost, which was collected from the Loushan sewage treatment plant in Qingdao, Shandong Province, China. The total weight of the experimental material was about 300 kg, where the sludge, backmix and peanut shells were fully mixed at a ratio of 3:1:1 (*w*/*w*/*w*). The physicochemical properties of the raw materials are shown in Table S1. The biochar material was tree leaves collected from Shandong Academy of Sciences, which were first washed with distilled water to clean the surface impurities, dried in an oven at 70 °C for 10 h, then crushed into sample pieces. The crushed samples were weighed and placed into crucibles with lids, which were placed in a muffle furnace at the temperature of 550 °C for anaerobic pyrolysis and maintained for 3 h after reaching the final temperature. After the furnace temperature dropped to room temperature, the samples were taken out and passed through a 100-mesh sieve to obtain the biochar, the production rate of which was about 39.3–40.0%.

The experiment was conducted in the laboratory with four composting treatments: ordinary compost (CT), compost with hyperthermophiles (HT), compost with hyperthermophiles and 2.0% biochar (HT2C) and compost with hyperthermophiles and 5.0% biochar (HT5C). Composting was conducted in four rectangular plastic containers (1.2 m × 0.6 m × 1.2 m) for 66 d, turning the compost over every 3 d. During the composting period, the temperatures of the upper, middle and lower parts of the pile were measured at noon every day to obtain the average value as the pile temperature.

### 2.2. Sample Collection

Samples were taken from the compost pile on the 0th, 2nd, 5th, 7th, 9th, 15th, 28th, 36th, 42th and 66th days. The samples were mixed thoroughly and then divided into three equal parts, of which one was kept in a refrigerator at 4 °C for pH, moisture, organic carbon, ammonium, electrical conductivity (EC) and germination index (GI) measurement during the composting process; another part was freeze-dried, sieved through 100 mesh and stored in a refrigerator at −20 °C for later extraction of DNA and determination of ARGs; and the remaining part was stored in a refrigerator at −80 °C for the detection of the bacterial community.

### 2.3. Real-Time Quantitative PCR (qPCR)

Genomic DNA was extracted using the TIANamp Soil DNA Kit (TianGen, Beijing, China). The extracted DNA was stored at −20 °C for further analysis. A total of 28 ARGs and 3 MGEs were screened in the composted material using gene primers. Finally, nine ARGs were determined comprising four tetracycline resistance genes (*tetA*, *tetG*, *tetO* and *tetM*), three sulfonamide resistance genes (*sul1*, *sul2* and *sul3*) and two macrolide resistance genes (*ermB* and *ermF*). At the same time, the gene copies of *intI1* and 16S rRNA were determined. The primers for all the target genes are listed in Table S2.

### 2.4. 16S rRNA Gene High-Throughput Sequencing

16S rRNA gene high-throughput sequencing was performed using the Illumina NovaSeq platform. The 16S V3–V4 region was amplified using the primers 341F (5’-CCTACGGGNGGCWGCAG-3’) and 805R (5’-GACTACHVGGGTATCTAATCC-3’). According to the unique barcode of the sample, the pairing end sequence was allocated to the sample, and the barcode and primer sequence introduced in the library construction were removed. The raw data was then filtered using fqtrim (v 0.94). The alpha diversity was used to analyze the species diversity in the samples through five indicators, including Chao1, observed species, goods coverage, Shannon and Simpson.

### 2.5. Statistical Analysis

To show the differences between samples, the correlation coefficients and variance (ANOVA) were performed using SPSS 19.0 (IBM, Armonk, NY, USA). Heatmap analysis was performed with R3.5.1. Redundancy analysis (RDA) was conducted using CANOCO 4.5. Spearman’s correlation coefficient (*p* < 0.01) was used as the basis for network analysis.

## 3. Results and Discussion

### 3.1. Physicochemical Changes during Composting

**Figure 1:**
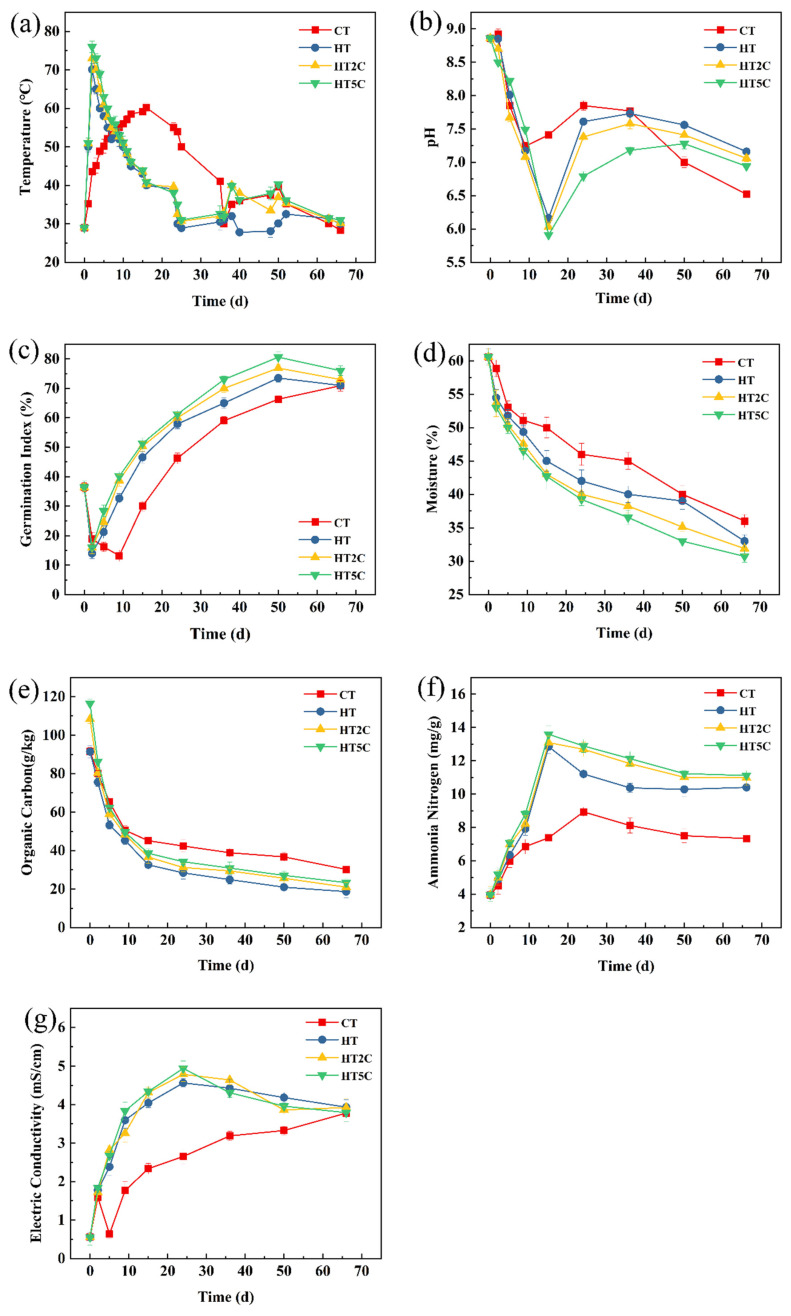
Physicochemical properties of composting: (**a**) temperature, (**b**) pH, (**c**) germination index (GI), (**d**) moisture, (**e**) organic carbon, (**f**) ammonia nitrogen and (**g**) electrical conductivity (EC).

The composting process can generally be divided into three stages according to the temperature changes, namely, thermophilic, mesophilic and maturation stages (Figure 1a). The maximum temperature in the HT5C treatment group reached 76.03 °C, which was higher than that of CT (60.18 °C), HT (70.12 °C) and HT2C (73.03 °C). Compared with the CT treatment, the addition of hyperthermophiles increased the composting temperature and shortened the warming time. Similarly, the addition of biochar also increased the composting temperature compared to the HT treatment, and the higher the addition proportion of biochar, the higher the reactor temperature.

During the composting process, the pH and GI first decreased, then increased and finally remained stable (Figure 1b,c). The biochar was alkaline but the pH of the treatments decreased with the addition of biochar. HT, HT2C and HT5C were weakly alkaline treatments and more suitable for the growing needs of microorganisms. The GI in the HT, HT2C and HT5C treatments were better than that of CT. The GI in four treatments at the end of composting were all above 70.0%, and the maturity of the compost products was up to standard. The moisture content and organic carbon kept decreasing throughout the composting process (Figure 1d,e). The moisture content in the different treatments was CT > HT > HT2C > HT5C. It was found that moisture content in HT5C decreased the fastest and was lowest at the end of composting, probably due to the higher temperature in the HT5C treatment. Throughout the composting treatment, the ammonia nitrogen content and EC showed a similar trend: increasing, then decreasing and finally stabilizing (Figure 1f,g). The ammonia nitrogen content was HT5C > HT2C > HT, suggesting that biochar addition promoted the fixation of ammonium nitrogen. The EC values of four composting treatments did not exceed 8 mS cm^−1^ throughout the composting process, which was within a reasonable range for microbial growth.

In the studied aerobic composting process, biochar with a porous structure could provide aerobic conditions for microbial growth and reproduction, as well as nutrients for microbial growth, which facilitated the growth and metabolism of microorganisms to generate heat [24], hence the addition of biochar increased the composting temperature compared to HT. The pH of the treatments decreased with the addition of biochar, which was mainly due to two reasons: on the one hand, biochar could adsorb more ammonia [25], making it difficult to dissolve, while ammonia in the CT and HT treatments without biochar was probably easier to dissolve and ionize to release OH^−^; on the other hand, biochar addition was more favorable for organic acid production [1,25]. The ammonium nitrogen content decreased in the later stage, which may have been due to the volatilization of ammonia, which was caused by turning the pile, and some of the ammonium nitrogen was converted to nitrate nitrogen or fixed by microorganisms as organic nitrogen [26]. In the studied aerobic composting process, organic matter was decomposed into CO_2_, H_2_O, NH_3_ and humus, which resulted in a continuous reduction of organic carbon content. In addition, the decomposition of organic matter by microorganisms produced mineral salts [27], which led to the rise of EC, but with the volatilization of ammonia caused by turning over pile and consumption of some organic salts (e.g., phosphate) by microbial, it gradually decreased [28,29].

### 3.2. Variation of ARGs and an MGE during Composting

**Figure 2:**
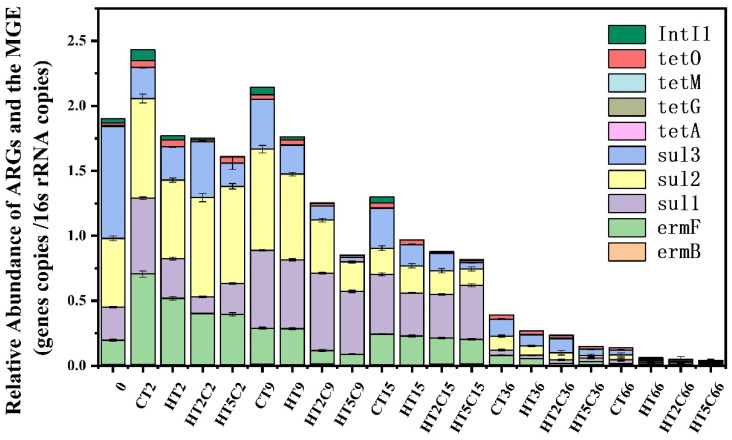
Changes in the relative abundance of ARGs in different compost piles. Bars denote the standard errors.

Nine ARGs and one MGE were detected in the composting material, where their relative abundances during the composting process are shown in Figure 2. At the beginning of composting, the relative abundance of *intI1* was highest in the CT treatment group among the four treatments. At the end of composting, the relative abundance of *intI1* in CT was 1.35, 4.64 and 4.81 times higher than that of HT, HT2C and HT5C, respectively. *IntI1* is abundant in the environment and also plays a crucial role in the transmission of ARGs [30]. The addition of hyperthermophiles and biochar inhibited the *intI1* and reduced the risk of ARGs diffusion in the compost [31,32]. Moreover, the addition of hyperthermophiles caused the temperature to rise, which might have damaged the plasmid, thus reducing the transmission of ARGs [32,33]. Biochar had a greater effect on *intI1*, but there was little difference in the removal of *intI1* between the different addition amounts of biochar (Table 1). Overall, the addition of hyperthermophiles and biochar during composting controlled *intI1*.

During the composting process, the relative abundance of total ARGs decreased gradually. After the composting, the relative abundance of total ARGs significantly reduced to 0.138, 0.063, 0.049 and 0.042 copies/bacterial cell for CT, HT, HT2C and HT5C, respectively (Figure 2). Especially in the middle and late stages of composting, the relative abundances of the ARGs in the four groups decreased significantly. The relative abundances of most ARGs decreased by the end of day 66 compared with the beginning of composting, but *tetM* increased. At the end of composting, the removal rates of total ARGs reached 72.7, 80.6, 84.3 and 84.8% for CT, HT, HT2C and HT5C, respectively (Table S3). The removal of *sul1*, *sul2*, *sul3*, *tetA* and *tetO* was further significantly increased in the HT treatment group with the addition of hyperthermophile compared to CT. The removal of *tetA*, *tetG* and *tetO* was further significantly increased in the HT2C and HT5C treatment groups with biochar addition compared to HT (Table 1). After the composting, the total ARGs removal rate of HT2C was significantly higher than that of HT, but there was no significant difference in the total ARGs removal rate between HT2C and HT5C (Table S3). Composting is an effective way to reduce most ARGs, but the abundance of some ARGs remained unchanged, while others increased in the compost [13]. The removal of most ARGs was improved by adding hyperthermophiles compared with CT, indicating that the high temperature during composting facilitated the removal of ARGs [33,34]: on the one hand, the decrease in MGEs in the composting process led to the efficient reduction in ARGs [21]; on the other hand, the high temperatures killed most of the non-thermophilic bacteria carrying ARGs, thus bringing about a reduction in ARG abundances [31]. The porous nature of biochar, which decreased the rate of microbe contact, may have been the main reason for the reduction in ARGs due to adding biochar [14]. The addition of different proportions of biochar had no significant effect on the abundance of most ARGs, which was consistent with a previous study [31]. However, compared with HT5C, the removal rates of *ermB,tetG* and *intI1* were better in HT2C. Considered together, the addition of 2.0% biochar to the compost effectively reduced the ARGs. However, it should be noted that only three ratios of biochar (0, 2.0 and 5.0%), in combination with hyperthermophiles, were investigated in this study, and future studies should consider more testing of different ratios of biochar additions to explore more effective methods of removing ARGs.

### 3.3. Evolution of the Soil Microbial Community

**Figure 3:**
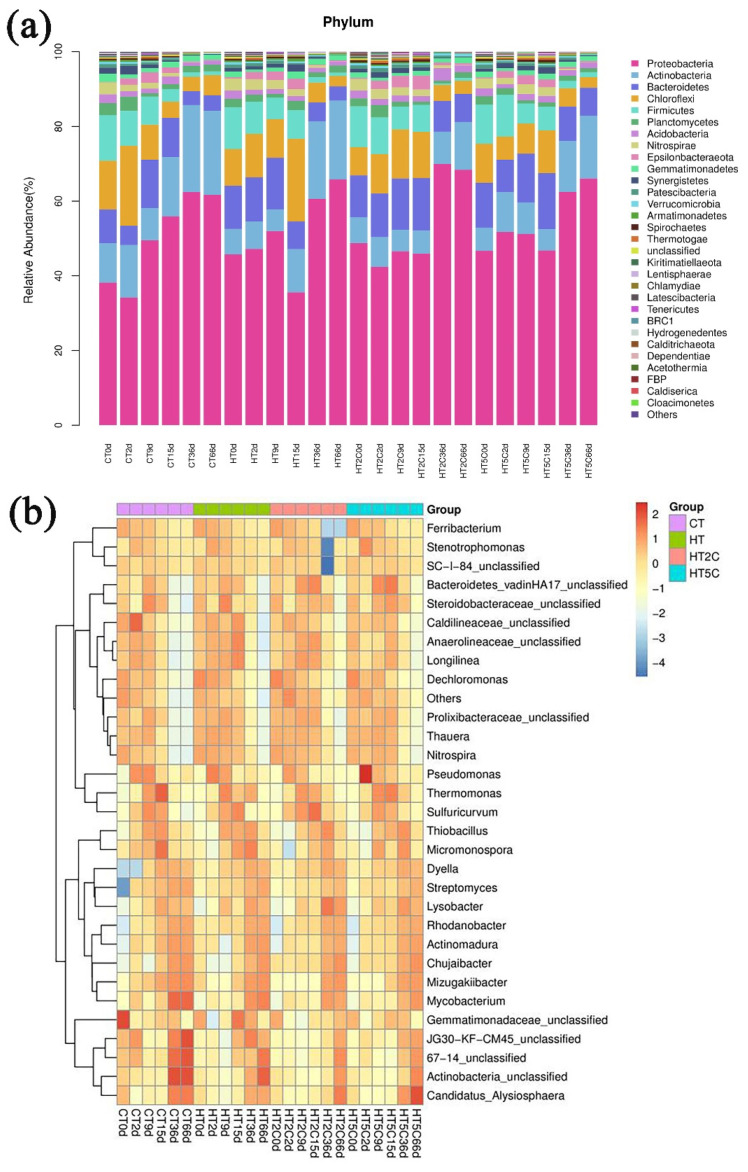
(**a**) Relative abundances of dominant phyla during composting. (**b**) The relative abundances of different bacterial genera in the different composting treatments.

The relative abundances of bacteria at the phylum level in all samples showed obvious changes in different composting stages (Figure 3a and Figure S1). Proteobacteria (46.0%), Bacteroidetes (11.0%), Firmicutes (15.0%), Chloroflexi (15.0%) and Actinobacteria (12.0%) were the most dominant phyla in raw material, accounting for 99.0% of the total bacterial 16S rRNA gene sequences. This was consistent with the distribution of the main bacterial community in the raw materials determined by previous studies [35]. 

In this study, Proteobacteria were the most abundant at the beginning of composting. At the end of composting on day 66, Proteobacteria increased by 61.7, 65.8, 68.4 and 66.1% in the CT, HT, HT2C and HT5C treatments, respectively. Meanwhile, Bacteroidetes, Firmicutes and Chloroflexi in four treatments showed a decreasing trend. In the CT, HT, HT2C and HT5C treatments, the relative abundances of Bacteroidetes decreased to 4.1, 3.8, 7.5 and 7.5%, respectively; Firmicutes decreased to 1.1, 0.9, 0.8 and 1.0%, respectively; and Chloroflexi decreased to 5.5, 2.8, 3.5 and 2.8%, respectively. The reduction in selection pressure led to significant variations in the bacterial community structure with composting [13]. Proteobacteria were highly tolerant and were an important source of ARGs [36], which played a key role in the composting rate and quality of the compost. Proteobacteria also had the function of breaking down small molecules, such as glucose [37]; therefore, the addition of hyperthermophile and biochar promoted the decomposition of organic matter and improved the composting process. The results showed that the addition of biochar inhibited the decline in Bacteroidetes. Compared with CT, the decline in Firmicutes in HT was less, which was related to its heat resistance [38]. Some researchers mentioned that Firmicutes is a group that probably carries and disseminates ARGs [38]. Therefore, the decline in Firmicutes abundance might explain the partial removal of ARGs in the composting process. The addition of hyperthermophiles further reduced the abundance of Chloroflexi, indicating that a high temperature reduces the relative abundance of Chloroflexi. After composting, the abundance of Actinomycetes increased in all four treatments, but the HT2C treatment had the least abundance of Actinomycetes. Actinobacteria have the function of carrying and spreading ARGs [39], which, on the one hand, explains the rebound of some ARGs at the end of composting [15] and, on the other hand, explains the better removal rate of some ARGs in HT2C. The main Actinomycetes that were identified during composting were Dyella and Streptomyces. Streptomyces are widely used in scientific and medical research because of their ability to produce antibiotics. It was obvious that the abundance of Streptomyces increased in the four treatments. The abundance of Actinomycetes was HT > HT2C > HT5C, indicating that biochar affected the reduction in Actinomycetes. 

The dynamic changes in dominant genera of the four treatments over time in the composting process are shown in Figure 3b and Figure S2. Under the selective pressure of hyperthermophiles and different concentrations of biochar, the microbial communities of the four treatments changed significantly compared to the initial bacterial community (0 d). The main bacterial communities in the composting materials were Thauera (6.2%), Dechloromonas (5.9%) and Ferribacterium (7.2%) (Figure S2). After composting, the abundances of the three bacteria were reduced to 0.3–0.5%, 0.1–0.2% and 0.0–0.1%, respectively, in the four treatments. Some bacteria, such as Rhodanobacter and Chujaibacter, were low in abundance at the beginning of the composting but gradually became more abundant in the compost. By the end of the 66 days of composting, the relative abundances of Rhodanobacter and Chujaibacter in the four treatments had reached 17.3–23.9% and 9.5–12.1%, respectively. However, the distribution of microorganisms in the same composting stage was different between the different treatment groups. Streptomyces and Actinobacteria also gradually became more abundant in the composting process, but the difference was that the enrichment degree of HT2C and HT5C in the treatments supplemented with biochar was lower than that of CT and HT, indicating that the addition of biochar had the effect of inhibiting Streptomyces and Actinobacteria. These results indicated that the addition of hyperthermophiles and biochar significantly affected the bacterial community structure and composition.

### 3.4. Relationships between the Bacterial Community, ARGs and the MGE

**Figure 4:**
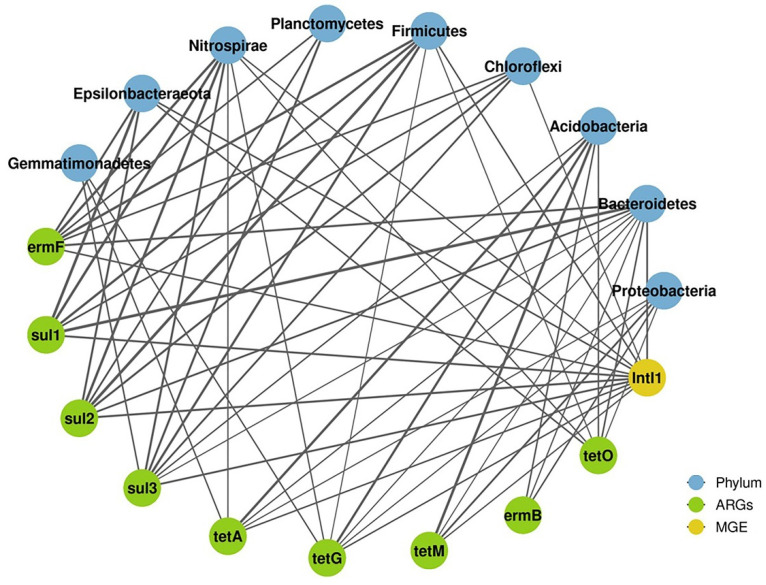
The correlation network represents the symbiotic relationship between ARGs and bacteria communities. Nodes with different colors represent different kinds of ARGs or bacteria.

The correlations among the bacterial community, ARGs and the MGE were studied using network analysis in the present study (Figure 4). We found that Firmicutes and Nitrospirae were strongly associated with most ARGs. Some ARGs had multiple potential host bacteria during the composting process. For example, *sul3* had significant positive correlations with Gemmatimonadetes, Nitrospirae, Planctomycetes, Firmicutes, Chloroflexi, Acidobacteria and Bacteroidetes. These ARG-related phyla lowered the abundances of ARGs, thereby bringing about the removal of *sul3*. Apart from *sul3*, there were a variety of potential host bacteria for all the other ARGs in the composting process. It was confirmed that the bacteria positively correlated with ARGs were considered to be host microorganisms of ARGs [40]. Therefore, bacterial genera in Firmicutes and Nitrospirae were found to be potential hosts for most ARGs, which was consistent with previous studies [14]. Variations in the abundances of ARGs were closely related to the bacterial community [41]. Certain microorganisms carry specific ARGs, resulting in similar abundance trends of ARGs [42]. Most of the potential hosts of ARGs were attached to Nitrospirae, Firmicutes, Acidobacteria and Bacteroidetes. These bacterial communities are regarded as important hosts for multiresistant ARGs [42]. The bacterial community, as the host bacteria of many ARGs, will affect the spread and transmission of ARGs [42]. Therefore, the identification of potential hosts through changes in bacterial communities plays a crucial role in the evolution of ARGs.

### 3.5. Conditions Affecting the Change of ARGs in Composting

**Figure 5:**
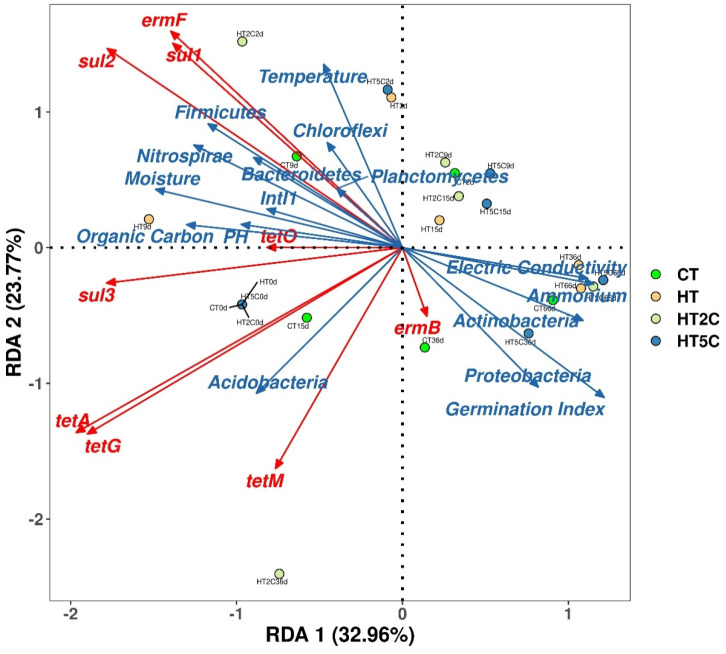
Redundancy analysis based on antibiotic resistance genes (ARGs), the mobile genetic element (MGE), microbial communities and environmental factors.

The relationships between the environmental factors, bacterial community, MGE and ARGs were assessed using RDA analysis (Figure 5). The environmental factors mainly included temperature, pH, moisture, electrical conductivity, organic carbon, ammonia nitrogen and germination index in this experiment. Among the environmental factors, pH and organic carbon were the main causes of the abundance variation in the ARGs and MGE. Meanwhile, pH and organic carbon were significantly correlated with nine ARGs and 16S rRNA (*p* < 0.01) (Table S4). In addition, organic carbon and pH was significantly correlated with the MGE (*p* < 0.01), suggesting that organic carbon and pH probably influenced the abundance of ARGs by affecting the MGE. The correlation between more kinds of environmental factors and ARGs and MGEs can be studied in the future, and the influence of various environmental factors on ARGs can be further explored.

In this study, RDA1 and RDA2 together explained 56.7% of the variation in the ARGs in the redundancy analysis, among which, the MGE was the major factor. Several studies showed that changes in the abundance of ARGs depend mainly on the potential host bacteria [40]. More research has shown that MGEs have a greater impact on ARGs than bacterial communities [43,44]. ARGs were directly influenced by *intI1*, and bacterial communities acted as indirect drivers. It was further confirmed that *intI1* was significantly correlated with the ARGs’ abundances (*p* < 0.01) using Pearson correlation analysis (Table S4), demonstrating that MGEs can influence the migration changes of ARGs through horizontal gene transfer (HGT) in different environments [45]. MGEs may increase the risk of ARGs spreading and significantly contribute to the variation in ARGs distribution [46]. In addition, integrons were destroyed during composting, and the presence of ARGs decreased when HGT was blocked. Therefore, the abatement of ARGs can be achieved by controlling the MGEs.

## 4. Conclusions

Adding hyperthermophiles and biochar in compost could effectively reduce ARG abundances. After composting, the relative abundances of total ARGs significantly reduced to 0.138, 0.063, 0.049 and 0.042 copies/bacterial cell for CT, HT, HT2C and HT5C, respectively. Among all of the factors considered in this study, *intI1* had the most important effects on the ARGs’ profiles. Treatments with different proportions of biochar added (HT2C, HT5C) had no significant effect on the abundance of ARGs, while other treatments were significantly different. However, HT2C showed better removal of *ermB*, *tetG* and *intI1* compared to HT5C. Considered together, adding 2.0% biochar combined with hyperthermophiles in compost could effectively control ARGs and MGEs. Future research should consider different ratios of biochar combined with hyperthermophiles to explore more effective methods of removing ARGs.

## Figures and Tables

**Table 1:** Comparison of removal rates of relative abundance of each ARG by adding different additives (over 66 d).

Treatments	*ermB*	*ermF*	*sul1*	*sul2*	*sul3*	*tetA*	*tetG*	*tetM*	*tetO*	*IntI1*
CT	13.8% a	96.2% c	87.5% c	93.0% c	96.0% b	59.3% d	88.7% c	--	23.1% d	96.9% c
HT	14.7% a	96.6% b,c	95.5% a	97.5% b	98.5% a	77.2% c	88.2% d	42.6%	60.0% c	97.7% b
HT2C	11.0% b	97.9% a,b	94.8% b	99.3% a,b	98.4% a	86.6% b	93.4% a	--	77.9% b	99.3% a
HT5C	7.9% c	98.5% a	95.3% a	99.5% a	98.8% a	92.2% a	91.6% b	--	80.1% a	99.3% a

The different lowercase in the table indicated significant differences between treatments at *p* < 0.05 level.

## Supplementary Materials

The following are available online at https://www.mdpi.com/article/10.3390/ma14185428/s1, Figure S1: relative abundance of composting bacterial community at the phylum level, Figure S2: Relative abundance of dominant genera during composting, Table S1: Physical and chemical properties of sludge and backmix, Table S2: PCR primers used in this study, Table S3: The removal rate of the relative abundance of total ARGs compared to the initial content in the raw material after composting (day 66) for the different treatment groups, Table S4: Spearman’s correlation coefficients among ARGs, MGE and environmental factors based on their relative abundances.
