# Supplementary material for: Biochar and Hyperthermophiles as Additives Accelerate the Removal of Antibiotic Resistance Genes and Mobile Genetic Elements during Composting
Source: Materials (Basel). 2021 Sep 19;14(18):5428. doi: 10.3390/ma14185428 (PMC8465662; doi:10.3390/ma14185428)
Supplement: Supplementary file 1 [file materials-14-05428-s001.zip › materials-1357488-supplementary.pdf]

materials

Biochar and Hyperthermophiles as Additives Accelerate the Removal of Antibiotic Resistance Genes and Mobile Genetic Elements during Composting

Yanli Fu, Aisheng Zhang, Tengfei Guo, Ying Zhu * and Yanqiu Shao *

Advanced Materials Institute, Qilu University of Technology (Shandong Academy of Sciences), Jinan 250014, China; f18766162009@163.com (Y.F.); zhangaisheng1207@163.com (A.Z.); gtf17663718837@163.com (T.G.)
* Correspondence: zhuyingee@163.com (Y.Z.); shaoyq@sdas.org (Y.S.); Tel.: +86-13969027728 (Y.Z.); +86-18325410320 (Y.S.)

Table S1. Physical and chemical properties of sludge and backmix.

Material	Moisture content（%）	pH	Organic carbon（g/kg）	Ammonium nitroge（mg/g）
Sludge	62.3	8.7	74.2	2.8
Backmix	40.0	7.4	30.2	7.3

Table S2. PCR primers used in this study.

Gene Name	Forward Primer	Reverse Primer	Annealing Temperature
tetA	GCTACATCCTGCTTGCCTTC	GCTACATCCTGCTTGCCTTC	59°C
tetG	GCACGCTGGTTTGGCTACA	TGGCTGTGATTAGTCTCCTTGA	54°C
tetO	ACGGARAGTTTATTGTATACC	TGGCGTATCTATAATGTTGAC	51°C
tetM	ACAGAAAGCTTATTATATAAC	TGGCGTGTCTATGATGTTCAC	56°C
sul1	CGCACCGGAAACATCGCTGCAC	TGAAGTTCCGCCGCAAGGCTCG	60°C
sul2	TCCGGTGGAGGCCGGTATCTGG	CGGGAATGCCATCTGCCTTGAG	59°C
sul3	TCCGTTCAGCGAATTGGTGCAG	TCCGTTCAGCGAATTGGTGCAG	55°C
ermB	GATACCGTTTACGAAATTGG	GAATCGAGACTTGAGTGTGC	55°C
ermE	GGTCGATCTTGACGGCTGG	GGTCGCGGAGGAAGTTCTG	55°C
intI1	GGCTTCGTGATGCCTGCTT	CATTCCTGGCCGTGGTTCT	60°C
16S rDNA	CCTACGGGAGGCAGCAG	ATTACCGCGGCTGCTGG	55°C

Table S3. The removal rate of the relative abundance of total ARGs compared to the initial content in the raw material after composting (day 66) for the different treatment groups.

Treatments	CK	HT	HT2C	HT5C
Total removal rates	72.7 %c	80.6 %b	84.3 %a	84.8 %a

The different lowercase in the table indicated significant differences between treatments at P < 0.05 level.

Table S4. Spearman's correlation coefficients among ARGs, MGE and environmental factors based on their relative abundances.

	PH	Temperature	Moisture	Electric Conductivity	Organic Carbon	Ammonium	Germination Index	Intl1
ermb	0.797**	-0.077	0.705**	-0.829**	0.811**	-0.697**	-0.467*	0.940**
ermF	0.836**	-0.033	0.817**	-0.875**	0.916**	-0.809**	-0.508*	0.986**
sul1	0.753**	-0.115	0.796**	-0.848**	0.931**	-0.776**	-0.430*	0.946**
sul2	0.732**	-0.264	0.784**	-0.892**	0.918**	-0.763**	-0.411*	0.943**
sul3	0.731**	-0.265	0.799**	-0.885**	0.915**	-0.778**	-0.407*	0.938**
tetA	0.709**	-0.261	0.781**	-0.848**	0.921**	-0.766**	-0.360	0.939**
tetG	0.801**	-0.144	0.828**	-0.926**	0.933**	-0.815**	-0.504*	0.908**
tetM	0.666**	0.031	0.604**	-0.786**	0.609**	-0.617**	-0.589**	0.934**
tetO	0.809**	-0.010	0.863**	-0.907**	0.950**	-0.836**	-0.566**	0.947**
16srRNA	0.647**	-0.377	0.745**	-0.820**	0.899**	0.718**	-0.284	0.976**
IntI1	0.724**	-0.107	0.726**	-0.895**	0.763**	-0.724**	-0.570**	1

* Significant at P < 0.05.

** Significant at P < 0.01.

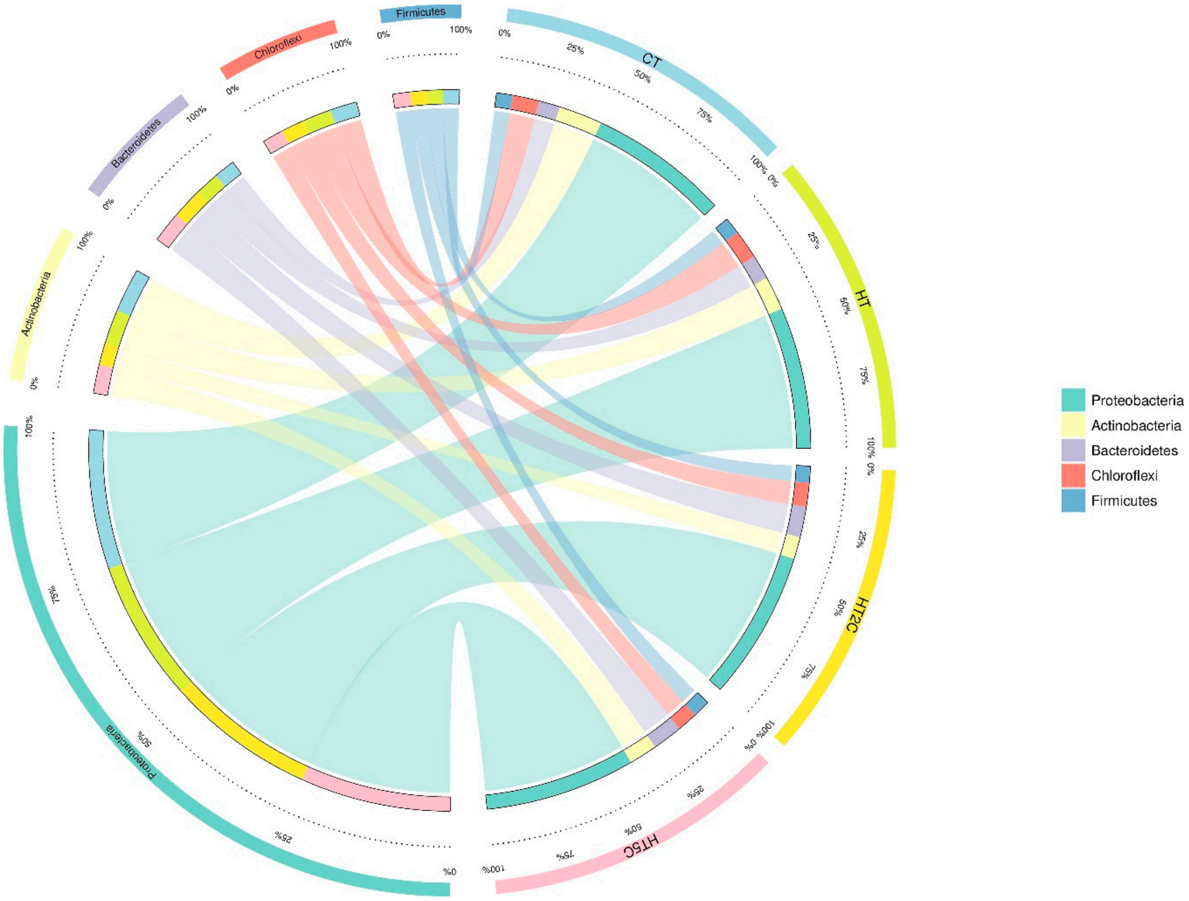

Figure S1. relative abundance of composting bacterial community at the phylum level.

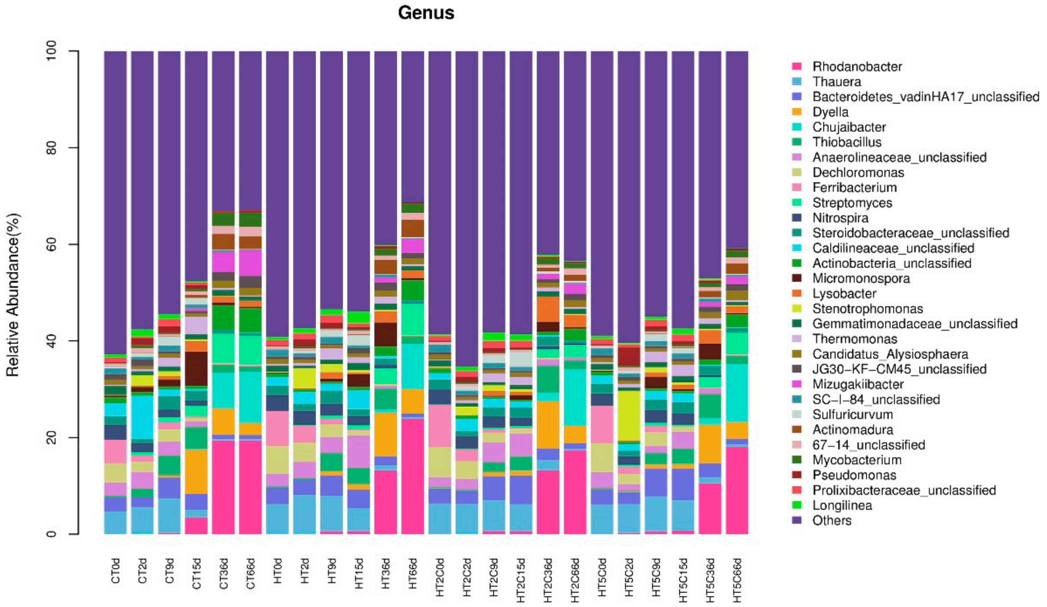

Figure S2. Relative abundance of dominant genera during composting.

© 2020 by the authors. Submitted for possible open access publication under the terms and conditions of the Creative Commons Attribution (CC BY) license (http://creativecommons.org/licenses/by/4.0/).
